# Metal Ions Fortified Tannin-Furanic Rigid Foam: The Impact on the Uniformity and Mechanical Performance

**DOI:** 10.3390/ma18030585

**Published:** 2025-01-27

**Authors:** Yang Yang, Haizhu Wu, Jun Zhang, Fajian Li, Bertrand Charrier, Hisham Essawy, Antonio Pizzi, Xiaojian Zhou, Xinyi Chen

**Affiliations:** 1 Yunnan Provincial Key Laboratory of Wood Adhesives and Glued Products, Southwest Forestry University, Kunming 650224, China; yangyang082024@163.com (Y.Y.); wuhaizhu@swfu.edu.cn (H.W.); zj8101274@163.com (J.Z.); 2 Key Laboratory of Forest Resources Conservation and Utilization in the Southwest Mountains of China Ministry of Education, Southwest Forestry University, Kunming 650224, China; 3 Kunming Freewill Furniture Manufacturing Co., Ltd., Kunming 650000, China; shadow.lifa@163.com; 4 CNRS, Institut des Sciences Analytiques et de Physico-Chimie pour lEnvironnement et les Matériaux-Xylomat, University of Pau and Pays de l’Adour, UMR5254, 40004 Mont-de-Marsan, France; bertrand.charrier@univ-pau.fr; 5 National Research Centre, Department of Polymers and Pigments, Cairo 12622, Egypt; hishamessawy@yahoo.com; 6 LERMAB, University of Lorraine, 27 rue Philippe Seguin, 88000 Epinal, France; antonio.pizzi@univ-lorraine.fr

**Keywords:** tannin, furfuryl alcohol, tannin-furanic rigid foam, metal ions, mechanical properties

## Abstract

Tannin-furanic foams with excellent properties have attracted increasing interest due to their advantages such as easy preparation, light weight, and thermal insulation. However, unsatisfactory mechanical strength has limited the expansion of their applications. Herein, three different metal ions (Cu^2+^, Fe^3+^, and Zn^2+^) were chosen to enhance the properties of tannin-furanic foam prepared by mechanical stirring provoked a foaming approach. The positive effects originating from the complexation are attributed to the associated connection between tannin molecules and metal ions. The results indicated that the apparent performance was improved, resulting in even foam cell structures. The apparent densities for the tannin-furanic foam modified with metal ions were located in the range of 36.57–47.84 kg/m^3^, showing the feature of lightweight material. The enhanced mechanical strength was verified by the compression strength (0.097–0.163 MPa) and pulverization ratio (7.57–11.01%) of the modified foams, which increased by 56–163% and decreased by 61–73%, respectively, in comparison with tannin-furanic foam without the metal ions. Additionally, the thermal conductivity of the modified tannin-furanic foams was in the range of 0.0443 to 0.0552 W/m·K. This indicates that they inherited the excellent thermal insulation typically associated with tannin-based foams. Interestingly, higher mechanical performance was obtained by comparison with other bio-sourced foams even with similar densities. In summary, by introducing only a small amount of metal ions, the foam performance was greatly improved, with a moderate cost increase, which reflects a good development prospect.

## 1. Introduction

Foam materials, normally including polyurethane (PU), polystyrene, and phenol–formaldehyde (PF) foams, have been wildly utilized in construction because of their exceptional properties such as light weight, thermal and sound insulation [1,2,3,4]. However, their inherent defects, for example, their high flammability for PU and polystyrene foams, and high brittleness for PF foam, along with their petroleum-based resources, have been encouraging researchers to suggest new foam formulations [5]. Several representative paths have been proposed: (i) A modifier is added while maintaining the original foam preparation formula to enhance their flame resistance and/or mechanical performance [6,7,8,9]; (ii) Foam materials with target properties (flame resistance and/or mechanical performance) are prepared by replacing some starting materials. For instance, lignin has served as a starting material to totally substitute petroleum-based polyols to prepare PU foams [10,11]; (iii) Foams with the desired performance are prepared by designing a novel formulation with high bio-mass content [12,13]. Typically, the third type tends to be of great interest to researchers.

A novel formulation of tannin-furanic foam product with over 90% bio-mass content has stimulated wide interest since it was first reported by Pizzi and his co-workers [14]. Its preparation is convenient, and it only needs the mixing of the ingredients directly by following a fixed order at room conditions. The self-polymerization of furfuryl alcohol and the co-reaction of furfuryl alcohol and tannin can occur in the presence of an acidic catalyst, forming a three-dimensional foam structure. It can also present comparable properties to some commercial counterparts, such as PU foams [15,16], polystyrene foams [17,18], and PF foams [19,20], in terms of the light weight (can be lower than 50 kg/m^3^), thermal insulation (as low as 0.045 W/m·K), and flame retardancy (LOI value is close to 30.0%).

Its boundedness (non-sustainable blowing agent and limited mechanical property), nevertheless, especially in typical tannin-furanic formaldehyde formulation, has gradually attracted more attention. Researchers have continually improved the tannin-furanic foams, considering the aspects of formula and structure. Glyoxal and glutaraldehyde [21], soybean protein isolate [22], lignin [13] and fossil resources (PEG-400) [21] have been reported to replace formaldehyde or employed as useful additives to enhance the tannin-furanic foams. The updated tannin-furanic foams have been advanced for the common goal of substituting some commercial foams with good comprehensive properties.

It should be noted that the traditional biomass tannin-furanic foam uses volatile organic compounds (VOCs) as foaming agents, such as diethyl ether (DE) [5,21,22,23] and azodicarbonamide (AC) [24]. Considering that the release of VOCs into the air affects the environment and human health, the mechanical agitation foaming method has been proposed and successfully applied, allowing the avoidance of the involvement of chemical foaming agents [25,26]. It provides significant value to the development of green foaming processes.

However, a fatal defect of tannin-furanic foams that cannot be ignored is the high brittleness (limited mechanical property), leading to a broad limitation of applications [22,26]. Varil [13] modified the tannin-furanic foam by adding lignin, which makes the foam almost 10 times stronger. The addition of a small amount of nanocellulose (CNF) [23] and wood cellulosic fibers [27] to the foam formula provides the foam a smaller pore size and improves compression strength. In addition, the double layer structure [26] has also been successfully developed to further improve the mechanical properties of the foam. Therefore, supposing a denser network spatial structure can be constructed in the foam material, the apparent morphology of the foam will most likely improve, allowing the obtainment of more uniform cell pores, and consequently, enhanced mechanical properties of the foaming material.

Encouragingly, some reports have illustrated that the hydroxyl groups on the B ring of condensed tannin (CT) show high affinity to various transition metal ions including iron ions, with the formation of a stable five-member ring [28,29]. Namely, tannin is able to complex with most trivalent metal ions when the two adjacent phenolic hydroxyl groups coordinate with the metal ions in the form of negative oxygen ions, resulting in the formation of a stable five-membered cyclic chelate structure [30], such as Fe^3+^, Al^3+^, Cr^3+^ and so on. A study based on this theory [31] has shown that a robust and 3D superhydrophobic composite sponge can be facilely prepared by the stable coating of multiple-walled carbon nanotubes (MWCNTs) onto melamine sponge (MS) using polyphenol-Fe^3+^ complexes as a low-cost and efficient interaction medium. In addition, tannin can not only interact with trivalent metal ions, but many researchers have also studied metal ions in other valence states and found that, for example, Cu^2+^ and Fe^2+^ are effective. Based on the complexation of catechol and pyrogallol structures with metal ions, it is of great significance to apply metal ions to the modification of tannin-furanic foams.

Moreover, furfuryl alcohol (FA), as another substrate of tannin-furanic foams, is generally activated in the presence of organic acids (*p*-toluenesulphonic acid) [5,22,26,27,32,33], acid zeolites (HY, HZSM-5) and Lewis acid (I_2_, SnCl_4_, TiCl_4_) [34]. Cesano et al. [34] proposed, for the first time, ZnCl_2_ as an acid promoter for FA polymerization at 60–70 °C to obtain porous ZnO–carbon composites. The polymerization of furfuryl alcohol carried out using ZnCl_2_ or CuCl_2_ as Lewis acid activators was investigated by exploring various synthesis parameters in order to produce activated carbons with different porosities and metal loads [35]. These studies have become the basic theory of our targeted modification approach for enhancing the overall properties of tannin-furanic foam.

The undesired mechanical strength of the classical tannin-furanic foam is possibly due to the inadequacy of crosslinking between tannin and furfuryl alcohol, resulting from the steric hindrance effect of tannin structure and its limited active sites. Actually, a large number of active groups such as phenolic hydroxyl groups in tannin molecules did not take part in the cross-linking. Herein, the possibility of metal ions upgrading the typical tannin-furanic foam preparation strategy was proposed, which can simultaneously replace the blowing agent using mechanical stirring instead, promoting the phenolic hydroxyl group’s participation in the crosslinking. Typical improvements have been attained, including (i) a chemical agent containing metal ions potentially acting as a Lewis acid activator to promote FA polymerization, and (ii) the catechol and pyrogallol structures on the tannin molecules being capable of electrostatic binding and complexation reactions with metal ions to achieve a stable combination with metal ions. Based on this, the possibility of metal ions upgrading the typical tannin-furanic foam preparation strategy was proposed, which can simultaneously replace the blowing agent, using mechanical stirring instead, which is expected to improve the mechanical properties. The as-obtained foams were systematically investigated in terms of apparent densities, micro-structure, and mechanical performance, etc. Interestingly, the upgraded tannin foams exhibited a lower density but over 2 times the compression strength measurements by comparison with the control group (without metal ions). The possible enhanced mechanism by metal ions was also proposed.

## 2. Materials and Methods

### 2.1. Materials

Commercial mimosa-condensed tannin extract (Acacia tree, its main components include 82% flavonoid monomers and oligomers, 8–12% of polymeric and monomeric carbohydrates, 4–6% residual water and 2–4% Others (inorganics) [22]) was purchased from TANAC S/A (R. Torbjorn Weibul, Montenegro, Brazil). Furfuryl alcohol (AR, C_5_H_6_O_2_, 98%), formaldehyde (37% in water), FeCl_3_, CuCl_2_, and C_4_H_6_O_4_Zn were purchased from Shanghai Macklin Biochemical Technology Co., Ltd., Shanghai, China. Tween 80 (CP) and *p*-toluenesulfonic acid (*p*-TSA, AR, ≥98.5%) were obtained from Sinopharm Chemical Reagent Co., Ltd., Shanghai, China. The distilled water utilized in all experiments was prepared in the laboratory. All other chemicals were used without further purification.

### 2.2. Preparation of Tannin-Furanic Foams

**Scheme 1:**
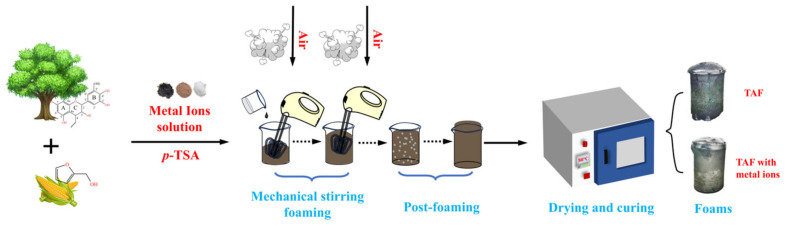
Illustrative diagram for preparation of tannin-furanic foams.

Initially, tannin and furfuryl alcohol were mixed and stirred with a whisk (Bear, DDQ-B02L1) at a low speed (200–300 RPM) for 1 min to obtain a homogeneous precursor. Then, the distilled water or/and metal ions solution were added according to the formulations listed in Table 1 to obtain a homogeneous mixture via stirring for 1–2 min. Thereafter, Tween-80 as a foam stabilizer and formaldehyde as a cross-linker were introduced into the former mixture. Finally, *p*-TSA (65% in water) was poured into the homogeneous mixture obtained from the previous step under the maximum speed (1000–1100 RPM) of the whisk used for 5–6 min to trap enough air for foaming the precursor until the resin started to heat up. The expanding liquid foam was left at room temperature (20–25 °C) for 2 h more (post-foaming process). After that, the tannin foams were moved to an oven heated to 80 °C for overnight solidification to obtain the final products. The foams were symbolized as TAF, TAF-Cu-0.6, TAF-Cu-0.9, TAF-Cu-1.2, TAF-Fe-0.6, TAF-Fe-0.9, TAF-Fe-1.2, TAF-Zn-0.6, TAF-Zn-0.9, and TAF-Zn-1.2, respectively, according to the types of metal ions and addition amounts. The foam preparation is simplified in Scheme 1.

### 2.3. Foam Characterizations

The foam products including control tannin-furanic foam (TAF) and tannin-furanic foams modified by metal ions (TAF-Cu-0.6, TAF-Cu-0.9, TAF-Cu-1.2, TAF-Fe-0.6, TAF-Fe-0.9, TAF-Fe-1.2, TAF-Zn-0.6, TAF-Zn-0.9, and TAF-Zn-1.2) were stored at ambient conditions (12–20 °C, 40–60% relative humidity (RH)) for at least 2 days prior to final characterization.

The apparent density (kg/m^3^) was calculated as the ratio of weight to geometric volume of a specimen with a size of 50 × 50 × 50 mm^3^ according to the ASTM D1622-03 [36], using the following formula:

(1) $$\mathrm{Density}\;(\frac{\mathrm{kg}}{m^{3}})\;=\frac{m\;(g)}{v\;({\mathrm{mm}}^{3})}\times 10^{6}$$

where m is the weight of the foam sample and v represents the volume of foam specimens. The average density was obtained from five repeated measurements.

According to GB/T 8813-2020 [37], a “Rigid cellular plastics-Determination of compression properties”, the compression strength of a foam sample was obtained using a universal mechanical testing machine (AG-50KN, Shimadzu, Japan) by loading with 50 kN. A foam sample with a size of 50 × 50 × 30 mm^3^ was positioned on the disc-like working surface, and the fixed crosshead began the compression of the foam specimen at a speed of 2 mm/min until the foam was completely destroyed. The final compression strength is defined as the maximum force before 10% foam deformation divided by the compressed surface area. The final compression strength was calculated by the following equation:

(2) $$\mathrm{Compression}\;\mathrm{strength}\;(\mathrm{MPa})\;=\frac{F_{\max}\left(N\right)}{S({\mathrm{mm}}^{2})}$$

where F_max_ is defined as the maximum force before 10% deformation, and S is the bottom area of the foam sample. In order to calculate the average compression strength, five different samples of each group foam were selected for the compressive strength test.

The standard of pulverization ratio was evaluated according to the Chinese National Standard GB/T 12812-2006 [38] “Rigid cellular plastics-Determination of friability”. The foam samples were cut into cubes with dimensions of 50 × 50 × 50 mm^3^ and the initial weight was recorded as M_0_. Subsequently, the foam sample was laid horizontally on 250 mm long sandpaper (400#) and a 200 g weight was placed above the foam. The foam sample was pulled by an applied force (automatic stringer) at a constant speed of 10 mm/s for 30 back and forth and the mass of the remaining foam was recorded as M_1_. The final pulverization ratio was calculated according to the following formula:

(3) $$\mathrm{Pulverization}\;\mathrm{ratio}\;(\%)\;=\frac{M_{0}-M_{1}}{M_{0}}\times 100\%$$

to calculate the average pulverization ratio, five replicated experiments were carried out.

The thermal conductivity was measured using a YBF-2 instrument (Dahua Ltd., Hangzhou, China). A foam sample with a radius of 50 mm and a thickness of 10 mm was covered with two copper plates, which were the same size as foam samples. The detailed operation procedure was reported in our previous work [26]. The thermal conductivity was measured using the following formula:

(4) $$\lambda =-\mathrm{mc}\frac{{2h}_{p}+R_{P}}{{2h}_{p}+{2R}_{P}}*\frac{1}{\pi R^{2}}*\frac{h}{T_{1}-T_{2}}*\left.\frac{\mathrm{dT}}{d\tau }\right|T=T_{2}$$

where λ denotes the thermal conductivity, m and c represent the mass and specific heat capacity of the copper plate at the bottom of the instrument, R (50 mm) and h (10 mm) represent the radius and thickness of the foam piece, and R_p_ (50 mm) and h_p_ (10 mm) denote the radius and thickness of the copper plate at the bottom, respectively. T_1_ − T_2_ is defined as the difference between the temperatures of the top and the bottom of the copper plate, and $\left.\frac{\mathrm{dT}}{d\tau }\right|T={\;T}_{2}$ denotes the cooling rate of the copper plate after exposure.

The foams were cut into specimens with dimensions of 10 mm × 10 mm × 10 mm, and the cell microstructure and morphology of the foams were observed using scanning electron microscopy (SEM, HITACHI SU8010, Hitachi Ltd., Tokyo, Japan) under an acceleration voltage of 15 kV. To improve the electrical conductivity, a thin gold coating was sputtered on the surface of each sample using a Leica EM ACE600 high-vacuum sputter coater.

TAF and TAF-Cu-0.9 foams were scanned and analyzed using a Bruker SkyScan 2211 (Bruker Corporation, Bremen, Germany) high-resolution integrated scanning analysis system. Foam samples were cut into cylinders of 20 mm diameter and 20 mm height for micro-CT to obtain high-resolution images with the optimized sample size at a scanning voltage of 30 kV and a current of 50 uA.

The thermal stability of the foam samples was estimated with a TG device (NETZSCH TG 209F3, NETZSCH Group, Selb, Germany). The samples were examined under a nitrogen atmosphere at a heating rate of 10 °C/min from room temperature to 790 °C, with about 5~8 mg of the sample per cycle.

X-ray photoelectron spectroscopy (XPS, Thermo Fisher ESCALAB 250Xi, Thermo Fisher Scientific, Waltham, MA, USA) was run using MgKa X-rays and a pass energy of 31.5 eV. The characteristic peaks of metal elements in the foam were observed using XPS and analyzed to ensure the participation of metal ions in the crosslinking with the tannin [39]. The XPS data were fitted using Avantage 5.9931 software.

## 3. Results and Discussion

### 3.1. Digital Photographs of Foams

**Figure 1:**
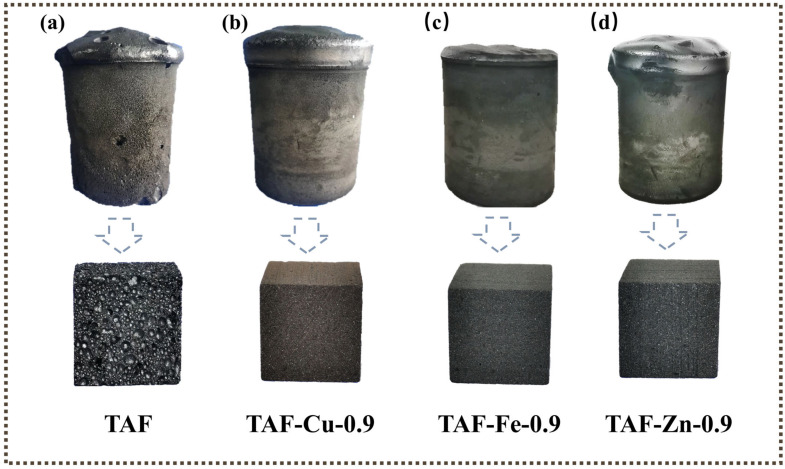
Digital photographs of control TAF (**a**) and metal ion-enhanced tannin-franic foams, including TAF-Cu-0.9 (**b**), TAF-Fe-0.9 (**c**), and TAF-Zn-0.9 (**d**).

Figure 1 exhibits digital photographs of tannin-based foams prepared by mechanical stirring without any blowing agent utilization. Some notable macroporous structures can be seen in the control group shown in Figure 1a, even revealing an uneven appearance. This phenomenon illustrates that the air bubbles are fused during the expansion of the foam volume. The most feasible reason is that partial solid structures, which are connected by the formaldehyde as a cross-linker and are involved in the formation of the foam cells, are easily breached by the heat-expanding gases. Conversely, a fine and uniform apparent structure was found for the metal ion-modified foams (Figure 1b–d). This result demonstrates that relatively stable foam cell structures reinforced by the metal ions were constructed, and in turn, they can entrap air bubbles more steadily so that the foam cells are prevented from fusing together. The modification efficiency by metal ions is proven through the improved apparent structural morphology.

### 3.2. The Morphology and Cell Size Distribution of the Foams

**Figure 2:**
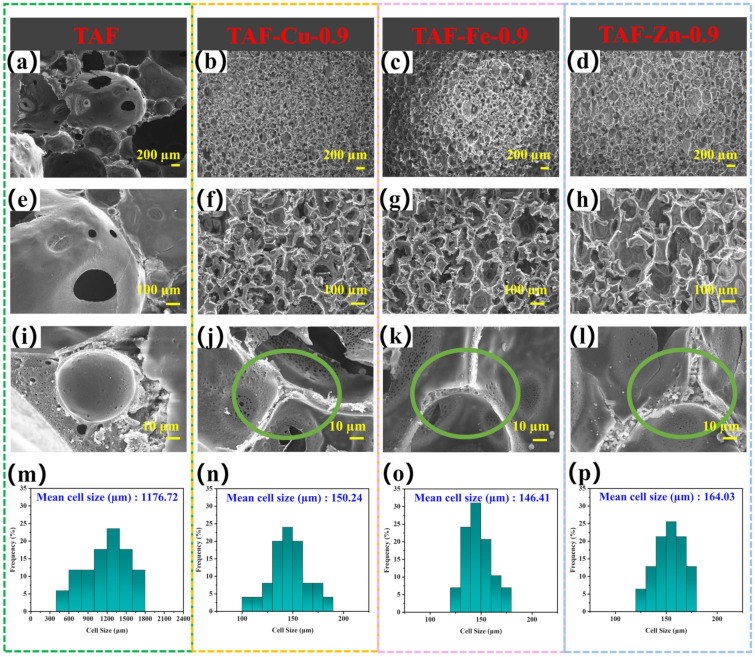
SEM micrographs and cell size distributions of tannin-based foams. SEM high-resolution micrographs of control TAF and metal ion-enhanced tannin-franic foams (TAF-Cu-0.9, TAF-Fe-0.9, and TAF-Zn-0.9). 30× magnification for TAF (**a**), TAF-Cu-0.9 (**b**), TAF-Fe-0.9 (**c**), and TAF-Zn-0.9 (**d**); 100× magnification for TAF (**e**), TAF-Cu-0.9 (**f**), TAF-Fe-0.9 (**g**), and TAF-Zn-0.9 (**h**); 1000× magnification for TAF (**i**), TAF-Cu-0.9 (**j**), TAF-Fe-0.9 (**k**), and TAF-Zn-0.9 (**l**); the cell size distribution of control TAF (**m**), TAF-Cu-0.9 (**n**), TAF-Fe-0.9 (**o**), and TAF-Zn-0.9 (**p**).

The microscopic morphology of foam specimens is shown in Figure 2 for control TAF, TAF-Cu-0.9, TAF-Fe-0.9, and TAF-Zn-0.9, from which the corresponding SEM micrographs can further explain the gained enhancement and modification effectiveness of metal ions. As can be seen from Figure 2a–i, the morphologies of the control TAF specimen and metal ion-enhanced tannin-franic foams (TAF-Cu-0.9, TAF-Fe-0.9, and TAF-Zn-0.9) show a great difference. Originally exhibiting a foam cell size with dispersed and large diameter (for the control group), they became more regular and delicate in the case of tannin-furanic foams modified with the metal ions at the same magnification. This is most likely one of the main reasons for the enhanced compression strength of the modified foams.

Furthermore, the triangular skeletal structure is a medium to connect adjacent foam cells, which could support the external loading, while the foam suffers from the compression. For the control TAF sample (Figure 2a,e,i), many irregular cellular defects can be detected despite the large area of these triangular connection areas. Moreover, some large sizes of “windows” can be observed on the cell wall. These defects and windows could destroy the integrity of the foam cells, which in turn could affect the overall strength of the foam. Unlike the control group, some differences can be seen: (i) the triangular cross region marked with green circles in Figure 2j,k,l clearly shows the relatively complete triangular skeletal structure of TAF-Cu-0.9, TAF-Fe-0.9, and TAF-Zn-0.9, with the stable skeleton structure considered one of the reasons for the increased compression strength of the foam; (ii) the relatively intact foam cells (even with some “small window groups” observed on it) contributed to the enhanced compression strength; and (iii), as shown in Figure 2n,o,p, TAF-Cu-0.9, TAF-Fe-0.9, and TAF-Zn-0.9 have smaller foam size diameters (around 150 μm), which means that a larger number of foam cells are distributed per unit area [26], providing a stronger supporting capacity, and thus a delayed collapse.

### 3.3. Micro-CT Images of Foams

**Figure 3:**
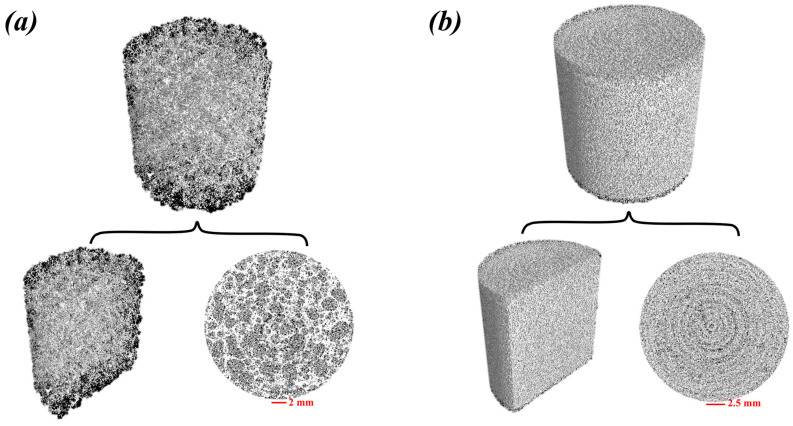
Micro-CT images of control TAF (**a**) and TAF-Cu-0.9 (**b**), revealing the detection images of the internal structures of the longitudinal section and cross-section of the sample.

X-ray micro-computed tomography (micro-CT) was utilized to examine the pore characteristics within the sample in more detail. Figure 3 shows the results of the micro-CT images for control TAF (a) and TAF-Cu-0.9 (b). It can be seen from the micro-CT displayed in Figure 3a that the overall apparent morphology of TAF is clearly rougher. This was further illustrated by the morphological features from the section inside the foam. The foam cell distribution is uneven and in conformity with the obtained results of SEM. In particular, it is further clearly observed that the size of the cells of TAF-Cu-0.9 (Figure 3b) is much smaller than that of TAF, and the cells are much more delicate and evenly distributed.

### 3.4. Analysis of X-Ray Photoelectron Spectroscopy

**Figure 4:**
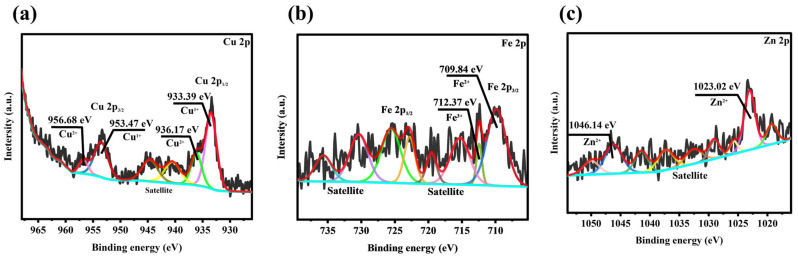
XPS spectra of the Cu 2p in TAF-Cu-0.9 (**a**), the Fe 2p in TAF-Fe-0.9 (**b**), and the Zn 2p in TAF-Zn-0.9 (**c**).

The XPS technique was utilized to investigate the possible crosslinking mechanism by metal ions and the corresponding spectra are shown in Figure 4. Some characteristic peaks appeared in Figure 4a–c, confirming Cu^2+^, Fe^3+^, and Zn^2+^ introduction, respectively. From the XPS spectra, we can observe the transition of the ion valence state, accompanied by strong satellite peaks [40]. From the XPS data (Figure 4a), the peak of Cu 2p at 933.39 eV is attributed to Cu^1+^, and the peak at 936.17 eV is attributed to Cu^2+^. The presence of Cu^1+^ indicates that the phenolic hydroxyl groups of the tannin undergo a redox reaction with Cu^2+^, which results in a reduction in Cu^2+^ due to transformation into Cu^1+^. This can be attributed to the formation of corresponding semi-quinones by tannin autoxidation [41,42]. Likewise, the peak of Fe 2p at 712.37 eV belongs to Fe^3+^ while the peak at 709.84 eV corresponds to Fe^2+^ [43,44]. The appearance of Fe^2+^ indicates that Fe^3+^ in the TAF-Fe-0.9 is partially transformed into Fe^2+^ because of the oxidization of phenolic hydroxyls to quinone [45,46]. Compared with Fe^3+^ and Cu^2+^, Zn^2+^ is less active [28], but a new peak of the Zn 2p peak (Figure 4c) appeared, indicating that Zn^2+^ is successfully involved in the synthesis of the foam. Hillis [47,48] showed that the addition of C_4_H_6_O_4_Zn to the reaction mixture at lower pH induced the reaction of catechol and flavonoid unit catechol B ring with formaldehyde. From this, it was speculated that the addition of the C_4_H_6_O_4_Zn involved more B-ring in the reaction and achieved further cross-linking of the tannin–formaldehyde network. In short, the addition of metal ions provokes the foam to form a dense cross-linked network with better mechanical properties.

### 3.5. Physical–Mechanical Properties of Foams

**Figure 5:**
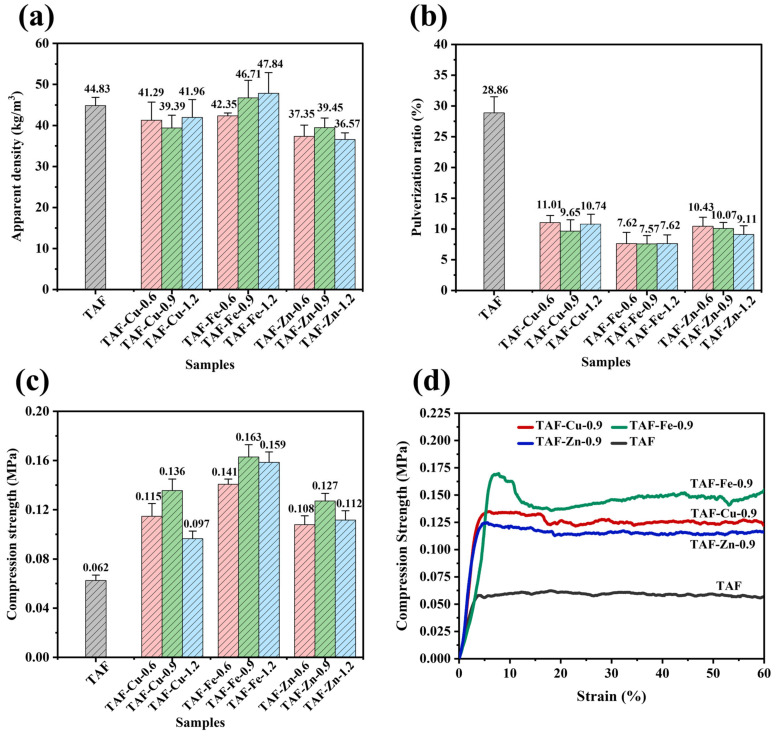
The apparent density (**a**), pulverization ratio (**b**), compression strength (**c**), and stress–strain curves (**d**) of control TAF and metal ion-enhanced tannin-franic foams (TAF-Cu-0.9, TAF-Fe-0.9, and TAF-Zn-0.9).

The apparent density, pulverization ratio, and compression strength of the control TAF and metal ion-enhanced tannin-franic foams (TAF-Cu-0.9, TAF-Fe-0.9, and TAF-Zn-0.9) are displayed in Figure 5. It can be seen that TAF (control) with a density of around 44.83 kg/m^3^ (Figure 5a), consistent with the common foams prepared by mechanical stirring, acquired an even lower density than the traditional tannin-furanic formaldehyde foams [22]. This result verified that the strategy of tannin-furanic foams with low density when prepared via mechanical stirring is a promising pathway. The modified tannin-furanic foams by metal ions with a density of 36.57–47.84 kg/m^3^ can be seen in Figure 5a. This phenomenon explains that the metal ion introduction did not increase the density of the foam, whereas it was even lower than the control group in most cases. This may be related to the function of the Lewis acid brought by metal ions, endowing sufficient stretching of the foam cells. Thus, a thinner cell wall structure was obtained (this can be verified from Figure 2).

The pulverization ratio of tannin-franic foams was investigated to further evaluate the positive role of inserted metal ions. The control TAF sample was 28.86% as summarized in Figure 5b, coinciding with the traditional tannin-furanic foam formulations and confirming the relatively poor cross-linking [26,33]. By comparison, the pulverization ratio of the modified foams with metal ions was around 7.57–11.01%, revealing a reduction of 61–73% (in Figure 5b). These results are promising, showing that more effective cross-linking took place between the foam components at the same density. The catechol and pyrogallol units present in the tannin are capable of undergoing electrostatic binding and complexation reactions [49], holding the responsibility for the reinforced cell structures.

The results of compression strength and stress–strain relations of foams are gathered in Figure 2c,d. It can be found that the compression strength of the modified foams by metal ions (TAF-Cu-0.9, TAF-Fe-0.9, TAF-Zn-0.9) was 56–163% higher than that of TAF foam (only 0.062 MPa). This was due to the fact that the surface morphology of the modified foams (TAF-Cu-0.9, TAF-Fe-0.9, TAF-Zn-0.9) is more delicate, along with smaller structures supporting the overall skeletal structure of the foams. This means there are more intact foam cells per unit of volume, increasing the effective number of cell pillars to prevent the collapse of the foam structure even under larger loads. This can clearly be observed in the apparent physical diagram of the foam (Figure 1). Moreover, the cell structures were enhanced, which was caused by the effective cross-linking between tannin and metal ions. This is in full accordance with the results of the pulverization ratio (Figure 2b).

### 3.6. Thermogravimetric Analysis

**Figure 6:**
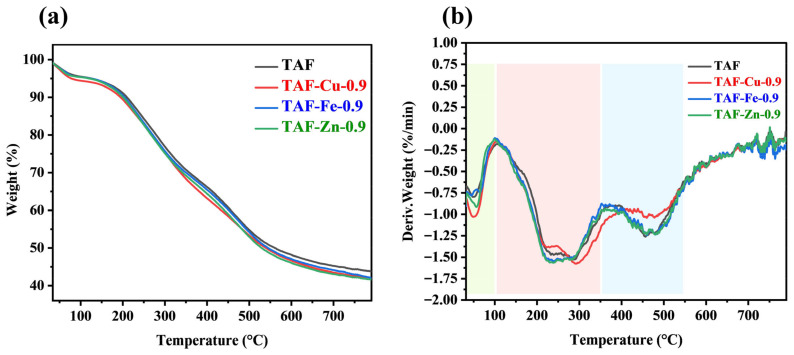
TGA curves (**a**) and DTG curves (**b**) of control TAF and metal ion-enhanced tannin-franic foams (TAF-Cu-0.9, TAF-Fe-0.9, and TAF-Zn-0.9).

The thermal stability of control TAF, TAF-Cu-0.9, TAF-Fe-0.9, and TAF-Zn-0.9 was investigated under a nitrogen atmosphere. The corresponding thermal decomposition traces of the foam structure are presented in Figure 6a, while their corresponding DTG appear in Figure 6b. The corresponding data are also summarized in Table 2. All foams exhibited three stages of degradation. The initial weight loss of TAF, TAF-Cu-0.9, TAF-Fe-0.9, and TAF-Zn-0.9 occurred at temperatures below 100 °C, which may be due to evaporation of entrapped water [27] and other volatiles (such as formaldehyde) [50]. Due to the decomposition of polymeric chains [27], TAF experienced a second weight loss at 282.8 °C, TAf-Cu-0.9 at 293.7 °C, TAF-Fe-0.9 at 241.7 °C and TAF-Zn-0.9 at 238.4 °C. This may be specifically related to the degradation of the foam stabilizing surfactant (Tween-80) [50]. In addition, it has been reported that the thermal decomposition temperature of the condensed tannins is about 300 °C, which may be one of the reasons for the weight loss [51]. For the final stage, most polymers degrade to form low-molecular-mass products, accounting for the main weight loss at that stage [27,32,52].

Although the modified foam and the original foam showed similar degradation stages, the addition of different metal ions had the same trend of effects on the thermal stability of the foam. Compared with the control group without metal ion addition, Cu^2+^, Zn^2+^, and Fe^3+^ reduce the thermal stability of foam slightly at high temperatures. For instance, when the pyrolysis temperature reaches 790 °C, the mass retention of TAF reaches 43.77%, while that of TAF-Cu-0.9, TAF-Fe-0.9 and TAF-Zn-0.9 attained 41.93%, 42.02% and 41.55%, respectively. This phenomenon was probably related to the increased thermal conductivity of foams in the presence of metal ions.

### 3.7. Thermal Conductivity

The thermal conductivity of control TAF and metal ion-enhanced tannin-franic foams (TAF-Cu-0.9, TAF-Fe-0.9, and TAF-Zn-0.9) is shown in Table 3. Similar to other rigid porous materials, the thermal conductivity of the tannin-furanic foams ranges from 0.032 to 0.050 W/m·K [53]. This means that tannin foams modified by metal ions did not change the features of thermal insulation of typical tannin-furanic foams. However, it can be seen that the thermal conductivity of tannin-furanic foam modified by metal ions became higher than that of control TAF foam, which is certainly related to the introduction of metal ions. The thermal vibration of the material was intensified due to the presence of metal ions, and the heat transfer efficiency between cells was elevated, and it became easier to generate solid heat transfer. The solid polymers containing metal ions may act as a dielectric skeleton for thermal and heat transfer in porous plastic foams [27]. Therefore, the thermal conductivity of the experimental group is higher than that of the control group at a similar foam density, but its thermal conductivity is still lower than that of most insulation materials made from forestry and agricultural waste resources [54,55].

### 3.8. Comparison with Other Literature

**Figure 7:**
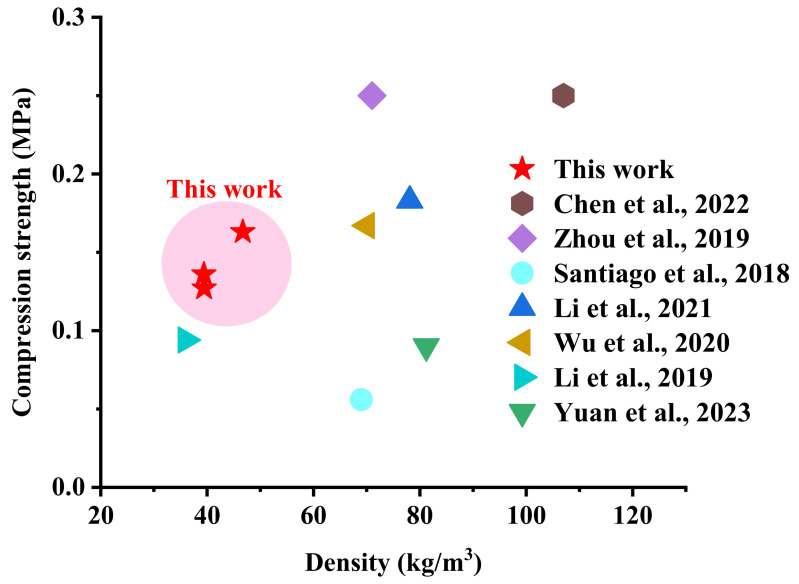
Comparison of density, compression strength, and cell pore size between metal ion-enhanced tannin-franc foams with other foams from literature [5,23,25,26,27,32,33].

A comparison was performed between metal ion-enhanced tannin-franic foams and other foams from the literature with respect to the apparent density and compression strength. The results are summarized in Figure 7. By contrast, tannin-furanic foams modified by metal ions exhibited a lower density, but presented a higher compression strength [5,23,25,26,27,32,33]. This indicates that the introduction of metal ions promoted the strengthening of the foam’s mechanical properties by forming a dense network structure with even apparent morphology. Interestingly, by just introducing a small amount of metal ions, the foam performance was greatly improved, and the cost was not increased greatly, which reveals a good development prospect.

## 4. Conclusions

In summary, this work reported an upgrading approach to modify tannin-furanic foams prepared by mechanical stirring foaming. Metal ions (Cu^2+^, Fe^3+^, Zn^2+^) can interact with the phenolic hydroxyl groups of tannin molecules, further compacting the intermolecular linkages of tannins by enhancing their intermolecular forces through chelation. In addition, salts containing target metal ions can be introduced to the formulations in small amounts, serving the synergistic effect of both Lewis acid and cross-linker. The resultant tannin-furanic foams displayed a uniform apparent performance and an even foam cell structure. Some other parameters could be promoted such as compression strength and pulverization ratio, which is due to the fact that the metal ions (Cu^2+^, Fe^3+^, Zn^2+^) and tannin build a robust three-dimensional network, making the network structure of the foam denser and more withstanding to greater pressure. The addition of metal ions (Cu^2+^, Fe^3+^ and Zn^2+^) significantly enhanced the compression strength of the foams, with the increase accounting for 56–163% compared with the TAF, and the pulverization ratio was correspondingly reduced by 61–73%. Interestingly, the acquired higher mechanical performance compared to other bio-sourced foams even with similar densities reveals another advantage. In addition, the modified tannin-furanic foams inherited the excellent thermal insulation associated with tannin-based foams. This study provides an effective strategy using a small amount of crosslinker to enhance the overall performance of tannin-furanic foams. This implies that the tannin-furanic foams modified by metal ions possess huge application potential in multiple cutting-edge fields, such as being the core component of new exterior wall insulation materials in the construction field and anti-collision materials in the transportation industry. They are expected to drive the innovation and upgrade of relevant industries and open a broader application landscape.

## Figures and Tables

**Table 1:** Formulations of the control group and upgraded tannin foams.

Samples	MT/g	FA/g	Tween-80/g	F/g	65 wt% *p*-TSA/g	Water ^1^/g	Metal Ions Solution ^2^/g
TAF	24	26	6	14.8	24	36	0
TAF-Cu-0.6						35.1	1.5
TAF-Cu-0.9						34.65	2.25
TAF-Cu-1.2						34.2	3
TAF-Fe-0.6						35.1	1.5
TAF-Fe-0.9						34.65	2.25
TAF-Fe-1.2						34.2	3
TAF-Zn-0.6						35.1	1.5
TAF-Zn-0.9						34.65	2.25
TAF-Zn-1.2						34.2	3

^1^ The water content in the final formulations was set to 36 g. ^2^ The mass fraction of metal ions to water (post addition) was fixed to 40 wt% (The reason for the metal ion aqueous solution with a concentration of 40% is that the preliminary experiments carried out in advance showed that metal ions aqueous solutions with too high or too low concentration could not achieve the ideal improvement effect on the performance of the foam materials. Based on this, we finally chose a metal ion aqueous solution with a concentration of 40%).

**Table 2:** Thermogravimetric data of control TAF and metal ion-enhanced tannin-franic foams (TAF-Cu-0.9, TAF-Fe-0.9, and TAF-Zn-0.9).

Samples	T_max_ (°C)			Residual Mass at 790 °C (%)
	Step 1	Step 2	Step 3	
TAF	49.5	282.8	456.7	43.77
TAF-Cu-0.9	50.0	293.7	461.5	41.93
TAF-Fe-0.9	42.1	241.7	457.3	42.02
TAF-Zn-0.9	57.8	238.4	460.9	41.55

**Table 3:** Thermal conductivity of control TAF and metal ion-enhanced tannin-franic foams (TAF-Cu-0.9, TAF-Fe-0.9, and TAF-Zn-0.9); the influence of metal ions on thermal conductivity of tannin foam was studied.

Samples	Thermal Conductivity/(W/m·K)
TAF	0.0329 (0.005)
TAF-Cu-0.6	0.0443 (0.007)
TAF-Cu-0.9	0.0486 (0.008)
TAF-Cu-1.2	0.0496 (0.010)
TAF-Fe-0.6	0.0492 (0.007)
TAF-Fe-0.9	0.0531 (0.004)
TAF-Fe-1.2	0.0552 (0.008)
TAF-Zn-0.9	0.0473 (0.009)
TAF-Zn-0.9	0.0503 (0.005)
TAF-Zn-0.9	0.0526 (0.006)

## Data Availability

The original contributions presented in this study are included in the article. Further inquiries can be directed to the corresponding authors.
